# A chain mediation analysis of the effects of psychological inflexibility, perception of threat, and behavioral adherence among refugees during crisis: The case of the COVID-19 pandemic

**DOI:** 10.1371/journal.pmen.0000462

**Published:** 2026-04-30

**Authors:** Martin Mabunda Baluku

**Affiliations:** Department of Educational, Social, and Organizational Psychology, School of Psychology, Makerere University, Kampala, Uganda; PLOS: Public Library of Science, UNITED KINGDOM OF GREAT BRITAIN AND NORTHERN IRELAND

## Abstract

Without known medications or vaccines for COVID-19, governments worldwide adopted the World Health Organization’s (WHO) preventive guidelines, which primarily focused on regulating physical and social interactions. Although the control measures effectively slowed the spread of the virus, their uptake in refugee settlements was hindered by the challenging living conditions within these settlements. This study examines the correlation of psychological factors, including psychological inflexibility, avoidance coping, perceived threat, and psychological trauma symptoms, with the uptake of COVID-19 preventive behavioral measures. It was hypothesized that avoidance coping, psychological trauma symptoms, and perceived threat mediate the association between psychological inflexibility and non-adherence. These assumptions were tested using data from 387 refugees from two large refugee settlements in Uganda. The results showed support for the hypothesized serial double mediation model. Overall, the results suggest that decreasing psychological inflexibility and consequently fostering adaptive coping could be essential for diminishing psychological trauma symptoms and stimulating accurate perception of risks and threats posed by crises and disasters, thus reducing non-adherence to preventive measures.

## Introduction

Governments worldwide imposed several preventive behavioral measures to slow the spread of coronavirus (COVID-19). These preventive behavioral measures included regulating hygiene practices and physical social interactions. The hygiene-promoting measures included frequent handwashing with soap or alcohol-based hand sanitizers, wearing face masks, and covering the mouth and nose when coughing or sneezing [1]. The behavioral measures, which tended to be the most stringent, included economic lockdowns (especially the closure of workplaces, shops, leisure and entertainment spaces, public and private transport, and stern regulation of the number of people in open markets), self-quarantine, curfews, and maintaining at least a 2-meter distance between people [1,2]. After the discovery of COVID-19 vaccines, vaccination was added to the preventive measures [1]. Whereas these measures effectively controlled the spread of the virus [2,3], there were variations in levels of adherence across different populations due to ecological and contextual factors [2,4].

Refugees’ behaviors, like other immigrants, are affected by acculturation. The level of acculturation influenced the trust in government communications, the health system, and the responses of refugees and immigrants to behavior change campaigns [5]. Moreover, refugees were likely to experience negative attitudes from host communities [6]. They were more likely to be considered coronavirus transmitters, given their mobile nature and the porousness of the Ugandan borders [7]. Such negative attitudes from the host communities affect refugees’ attitudes and behavior, likely leading to non-adherence to preventive behavioral measures.

The living conditions in refugee settlements in low-income countries were considered a possible impediment to implementing and observing COVID-19 control measures [8]. With over 1.35 million refugees, Uganda was among the top five refugee-hosting nations in the world before the emergence of the pandemic [7]. The majority of these refugees lived in poor and overcrowded settlements and makeshift structures with poor sanitation and hygiene [9,10], yet infections and mortality were higher in such contexts [11]. This situation increased the risk of contagion. Moreover, previous research shows that crowdedness is an antecedent of stress and problem behavior [12]. Hence, the likelihood of non-adherence could have been higher among refugees in crowded settlements. In addition, the language barrier means that refugees had great difficulty understanding public educational communications on COVID-19 and the preventive measures [9,13], further increasing the risk of non-adherence and susceptibility to contagion. Given the resource scarcity in many settlements, refugees often crowd at places where they can access essential social amenities such as water and medical care [14,15], thereby increasing social contact and the risk of spreading the virus.

Generally, refugees tend to have a history of traumatic experiences that have consequences on mental health [8,9,14]. The mental health challenges are likely to be a barrier to an accurate assessment of health threats and associated outcomes, consequently diminishing their adherence to preventive measures [8,9]. The current study focuses on the psychological states and attributes that may have contributed to non-adherence, thereby reducing the uptake of COVID-19 preventive behaviors among refugees in resource-constrained refugee settlements in a less developed country. Based on the provisions of the Acceptance and Commitment Therapy “ACT” [15,16]. The study postulates that psychological inflexibility and the associated avoidance coping could have affected the perception of the threat of COVID-19 and stimulated psychological trauma symptoms, resulting in lowered adherence to the preventive behavioral measures.

In the ACT model [15], psychological inflexibility predicts psychological dysfunction and constricts behavioral adjustment. Psychological inflexibility is conceptualized as the rigidity in psychological reactions to disturbing or unwanted internal events [17,18]. Psychological inflexibility comprises experiential avoidance, rigid attention, disrupted values, impulsivity, conceptualized self, and cognitive fusion [15,17], which facilitate the development of psychopathology and the experience of pain or suffering [19]. In this direction, previous studies have linked psychological inflexibility to psychological trauma and the development of Post-Traumatic Stress Disorder (PTSD) in various populations, such as military veterans [20–22], patients and caregivers [23,24], students [25,26], and refugees [27]. Similar effects were observed in COVID-19-related studies [28–30]. Moreover, reduced psychological inflexibility has been associated with positive behavior change and improved psychological health [22,31,32].

The above description of psychological inflexibility suggests that it is a concept that represents maladaptive tendencies. We posit that the maladaptive qualities associated with psychological inflexibility may have made it a potential antecedent of low adherence to preventive behavioral measures aimed at slowing the spread of COVID-19 among refugees. Particularly, when individuals are psychologically inflexible, their behavior tends to be rigidly controlled by avoiding unwanted or unpleasant thoughts, emotions, memories, sensations, and experiences rather than focusing on one’s values and goals [28, 20]. Consequently, these psychological reactions result in the development and persistence of behavioral dysfunctions, including behavioral adherence. In addition, psychological inflexibility influences how individuals respond and cope with challenges [33]. The avoidance mechanisms involved in psychological inflexibility are likely to breed maladaptive rather than approach-based coping strategies. This supposition is supported by empirical studies showing that psychological inflexibility tends to result in behavioral disengagement [34,35].

The role of psychological inflexibility in behavior disengagement or adjustment may be associated with its impact on the perception or evaluation of threats. The fear of COVID-19 was essential in how people responded to behavioral measures and was critical for mental health outcomes during the pandemic [36]. Psychological inflexibility influences perceptions under challenging situations. Inflexible attention, one of the core processes involved in psychological inflexibility, produces rigidity in thoughts and beliefs, thus affecting evaluation of the situation, problem, or threat. Therefore, psychological inflexibility was more likely to inhibit the perceptions of the risks associated with COVID-19, leading to lowered adherence [37].

Psychological trauma is one of the most common mental health problems in refugee populations. Moreover, COVID-19 elicited psychological trauma symptoms in the general public and specific groups, such as health service providers [38,39]. Extant research shows that psychological inflexibility is a strong antecedent of poor mental health outcomes and hence a determinant of higher psychological trauma symptom severity [20,40], which inhibits behavioral adjustment. Yet, psychological trauma can be exacerbated by stressful situations. By facilitating problem behavior and avoidance tendencies [41], psychological trauma negatively affects behavioral adjustment [42]. Therefore, perceived threat and psychological trauma symptoms could be underlying mechanisms through which psychological inflexibility and avoidance coping impact the uptake of preventive behavioral measures among refugee populations.

The current study aimed to assess whether the effects of psychological inflexibility on the uptake or adherence to COVID-19 preventive behavioral measures were mediated by avoidance coping, psychological trauma symptoms, and perceived threat. Based on the theoretical discussion above, I hypothesized that:

Hypothesis 1: Psychological inflexibility had positive effects on (a) avoidance coping, (b) psychological trauma symptoms, (c) non-adherence to preventive behavioral measures, and (d) negative effects on perception of threat of COVID-19.

Hypothesis 2: (a) avoidance coping, (b) psychological trauma symptoms, and (c) perception of threat of COVID-19 mediated the effects of psychological inflexibility on non-adherence to the preventive behavioral measures.

Hypothesis 3: The association between psychological inflexibility and refugees’ non-adherence to the preventive behavioral measures was serially mediated through:

a) avoidance coping and psychological trauma symptoms, and
b) avoidance coping and perceived threat.

## Methods

### Ethics statement

This paper uses date from the project “Investigating and Addressing COVID-19 Related Mental Health Challenges in Refugee Settlements and Host Communities in Uganda”. The study received ethical clearance from the Gulu University Research Ethics Committee (No. GUREC-2020–32). The study also received administrative clearance from the Office of the Prime Minister of the Government of Uganda. Ethical matters such as participants’ safety, welfare, confidentiality, autonomy, justice, and informed consent were adhered to in line with the Helsinki Declaration [43].

All participants were required to sign the formal informed consent form.

### Participants and procedure

To test the hypotheses, a survey was carried out among South Sudanese and Somali refugees residing in Uganda’s urban and rural refugee settlements. Participants were drawn from the refugee population living in the Kampala metropolitan area (including Kampala, Wakiso, and Mukono districts), which hosts most of the urban refugees in Uganda, and from Bidibidi refugee settlement in the West Nile region of Uganda. Bidibidi was considered the second-largest refugee settlement in the world, with over 270,000 refugees [44], mainly from South Sudan. Table 1 presents the demographic data, differentiated by urban and rural settlements.

**Table 1:** Participant characteristics by type of settlement.

Variable	Categories	Rural	Urban
Average age	In years (SD)	30.775 (9.438)	29.417 (8.316)
Average stay in Uganda	In years (SD)	4.247 (2.440)	5.147 (4.235)
Gender	Female	84	28
	Male	147	128
Country of origin	South Sudan	71	243
	Somalia	54	1
	Other	13	5
			

Data were collected during the lockdown (October 1^st^ – 31^st^, 2021), when all restrictions on social interactions and movement were still in force. Participants were recruited within the refugee settlements with the support of zonal and camp leaders, as well as other service providers, under strict observance of the guidelines against COVID-19 set by the Government of Uganda. The sample comprised 402 refugees, although only 387 participants (71.1% males, 28.9% females, 40.3% living in the urban settlement, and 59.7% living in the rural settlement) provided complete responses. The adequacy of the sample size was tested using the G-power v3.1 [45] sample size calculator. Accordingly, the minimum number of participants required for regression analysis with eight [8] antecedent variables at an effect size of 0.15, probability level of 0.01, and statistical power of 0.99 is 219. Hence, the number of responses used for this study is considered sufficient. The participants were primarily young people with an average age of 30.20 years (*SD* = 9.07, *Range* = 18 – 74 years). Participants had also lived in Uganda for a relatively long time (M = 4.61 years, *SD* = 3.31). Despite collecting this demographic information, we did not collect any information that could identify participants. Data on participant characteristics are summarized in Table 1. Since the participants were drawn from both urban and rural settings, a summary of the differences in the study variables by these living contexts are provided in Table 2.

**Table 2:** Differences in the study variables by type of settlement.

Variables	Differences by type of settlement						
	Settlement	*N*	*M*	*SD*	*SE*	*t*	*p*
Psychological inflexibility	Urban	156	3.54	1.48	.12	-1.46	.144
	Rural	231	3.76	1.40	.09		
Avoidance coping	Urban	156	3.25	.97	.08	-3.61	.000
	Rural	231	3.63	1.03	.07		
Psychological trauma symptoms	Urban	156	3.40	1.09	.09	-2.93	.004
	Rural	231	3.72	1.01	.07		
Perceived threat	Urban	156	2.89	1.61	.13	.55	.584
	Rural	231	2.79	1.82	.10		
Non-adherence	Urban	156	3.67	1.28	.10	-1.47	.143
	Rural	231	3.86	1.27	.08		

## Measures

The study used a survey questionnaire. The questionnaire was translated into the major languages spoken in the two refugee settlements: Somali and South Sudanese Arabic. The translation process followed the back translation procedure [46]. Native Somali and South Sudanese Arabic speakers translated the survey questionnaire from English. Different individuals translated the Somali and Arabic versions back into English. Both translators collaborated to resolve the issues identified during the back-translation process.

### Psychological Inflexibility

The Avoidance and Action Questionnaire “AAQ” [18] was used to assess psychological inflexibility, which examines the rigidity in the processes of dealing with undesirable internal experiences [47]. The questionnaire consists of seven [7] items that were measured on a 6-point scale (1 = not at all, 6 = very much). A sample item is “My painful memories prevent me from having a fulfilling life.” The questionnaire showed acceptable internal consistency (α = .78).

### Avoidance coping

The Brief COPE [48] was used to assess refugees’ usage of the avoidance coping strategy. This scale has been structured differently, including a two-factor structure, with the other being the approach coping strategy [33,49]. Avoidance coping dimension was measured using twelve [12] items, which assess several aspects of avoidance behavior such as denial, substance use, behavioral disengagement, and self-blaming. A sample item is “I’ve been giving up the attempt to cope” (responses ranging from 1 = Not at all to 6 = a lot). The scale showed acceptable internal consistency (α = .70).

### Psychological trauma symptoms

The revised Impact of Events Scale “IES-R” [50] was used to examine the presence of psychological trauma symptoms among the participants. The IES-R comprises 22 items assessing the presence of different psychological trauma symptoms. A sample item is “I had trouble falling asleep” (with responses ranging from 1 = Never to 6 = very often). This scale is considered to have good psychometric properties, therefore valid to use clinically and for research [51]. The IES-R had an impressive level of internal consistency in the present study (α = .81).

### Perceived threat

The study used the three items in Baluku [39] to assess participants’ perception of the threat of COVID-19. A sample item is “COVID-19 does not affect people of my age” (with responses ranging from 1 = totally disagree to 6 = totally agree). The items showed acceptable internal consistency (α = .70).

### Non-adherence to preventive behavioral measures

To assess adherence/ non-adherence, the questionnaire developed by Baluku, et al [39] was used. The questionnaire comprises 30 items that evaluate the extent to which individuals forgot to follow the COVID-19 preventive behavioral guidelines. A sample item is “How often do you have difficulty keeping a distance of at least two meters from people who do not belong to your household” (responses ranging from 1 = never to 6 = very often). The items showed high internal consistency (α = .90).

### Analytic approach

**Fig 1:**
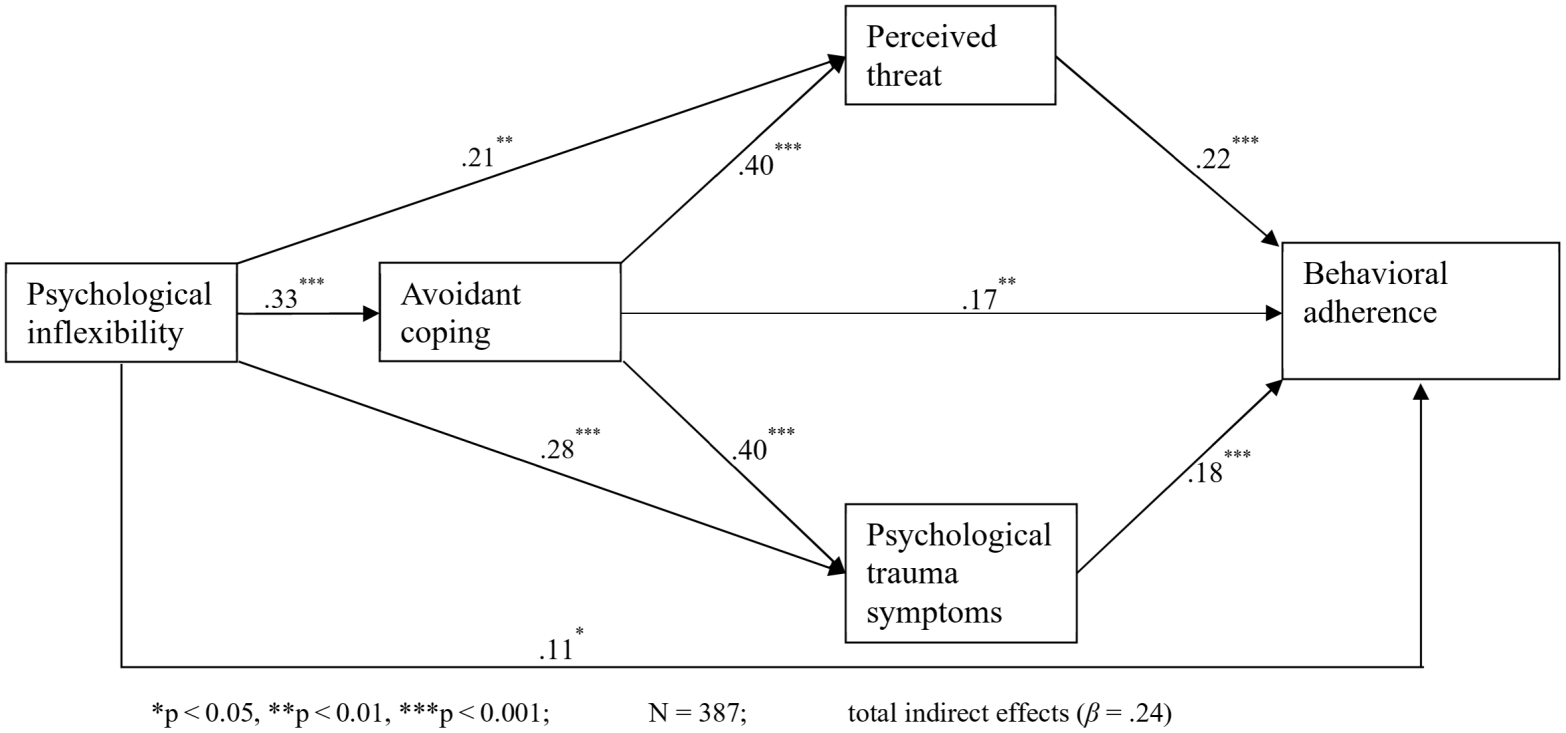
Path model of avoidant coping, perceived threat, and psychological trauma symptoms as mediators of effects of psychological inflexibility on non-adherence to the behavioral measures.

To test the hypothesized serial double mediation model (Fig 1), a sequential mediation analysis in PROCESS Macro [52] was used. Model 81 of the PROCESS Macro was applied since it simultaneously tests the effects of various mediator variables. We used Pearson Product-Moment Correlations (bivariate) to test whether the data meets the assumptions for mediated regression analysis. Demographic variables assessed in the study, including sex, age, type of settlement, and the number of years lived in Uganda, were not included in the regression model since they were not substantially correlated to the outcome variable. In addition, Bootstrapping at 5,000 and confidence intervals at 95% were applied in the regression analysis. I tested several assumptions for a multiple regression. First, it is assumed that multicollinearity can impact observed relationships among variables, especially if Variance Inflation Factor (VIF) values are greater than 5 and Tolerance (TOL) values are below.2 [53]. For the present study, VIF varied from 1.22 to 1.59. and the TOL varied from.63 to.83. Second, normality of data for all variables was assessed using the Shapiro-Wilk test, which revealed that the data were not normally distributed. However, the histogram and normal P-P plot of the regression standardized residual showed a normal distribution. Moreover, the skewness statistics in Table 4, considered the most important for normality, are within the recommended range of ±1 [54]. Therefore, the data can be considered normally distributed, and the significant Shapiro-Wilk test coefficients could have resulted from a relatively large sample size. Cook’s distance test did not reveal substantial issues concerning outliers, while the Durbin-Watson coefficient of 1.76 indicates there are no autocorrelations, hence satisfying the independence assumption. There were also no substantial concerns relating to linearity and homoscedasticity.

## Results

Means, standard deviations, and correlations among the study variables are presented in Table 3. Considering the descriptive statistics, the level of perceived threat of the COVID-19 virus was relatively high, while avoidance coping, psychological trauma symptoms, and non-adherence to the preventive behavioral measures was at a moderate level.

**Table 3:** Descriptive statistics, reliabilities, and correlations among variables.

	*M*	*SD*	*α*	1	2	3	4	5
1. Psychological inflexibility	3.67	1.44	.78	–				
2. Avoidance coping	3.48	1.02	.70	.46^***^	–			
3. Psychological trauma symptoms	3.59	1.05	.81	.46^***^	.52^***^	–		
4. Perceived threat	2.83	1.74	.70	-.34^***^	-.38^***^	-.27^***^	–	
5. Non-adherence	3.78	1.28	.90	.35^***^	.40^***^	.39^***^	-.37^***^	–

* p < 0.05, ** p < 0.01, *** p < 0.001; N = 387;

**Table 4:** Testing assumptions for multiple linear regression analysis.

Variable	Shapiro-Wilk Score		Skewness		Kurtosis		Collinearity statistics	
	*Statistic*	*p*	*Statistic*	*SE*	*Statistic*	*SE*	*TOL*	*VIF*
Psychological inflexibility	.960	.000	-.068	.124	-.775	.247	.699	1.430
Avoidance coping	.986	.001	.301	.124	-.262	.247	.630	1.588
Psychological trauma symptoms	.991	.015	.158	.124	-.267	.247	.665	1.503
Perceived threat	.862	.000	.540	.124	-.976	.247	.822	1.216
Non-adherence	.980	.000	-.106	.124	-.677	.247	–	–

Note: N = 387

Results of the chain mediation regression analyses are presented in Table 5. Concerning Hypothesis 1, psychological inflexibility was positively correlated to avoidance coping (*β* = .33, *95% CI* [.26,.40], *p* < .001), psychological trauma symptoms (*β* = .28, *95% CI* [.13,.28], *p* < .001), and non-adherence to the preventive behavioral measures (*β* = .11, *95% CI* [.01,.20], *p* < .05). Hence, hypotheses 1a, 1b, and 1c are supported. Moreover, psychological inflexibility was also positively correlated to the perception of the threat of COVID-19 (*β* = .21, *95% CI* [.13,.38], *p* < .001); thus, hypothesis 1d was not supported. Avoidance coping was positively related to psychological trauma symptoms (*β* = .40, *95% CI* [.30,.52, *p* < .001) and non-adherence to the preventive behavioral measures (*β* = .17, *95% CI* [.07,.35], *p* < .01). Surprisingly, avoidance coping was also positively associated with perceived threat (*β* = .28, *95% CI* [.30,.65], *p* < .001). In addition, both psychological trauma symptoms (*β* = .18, *95% CI* [.09,.35, *p* < .001) and the perception of threat of COVID-19 (*β* = .22, *95% CI* [.09,.23, *p* < .001) were positively associated with non-adherence to preventive behavioral measures.

**Table 5:** Regression results of the chain mediation analyses.

Predictors	*β*	*SE (HC4)*	*t*	*95% CI*	
				*[LL,*	*UL]*
*Model 1: Predicting avoidance coping (AC)*					
Psychological inflexibility (PI)	.33^***^	.04	9.32	[.26,	.40]
Model statistics	*R*^*2*^ = .21, *Adj R*^*2*^ = .21, *F*(HC4) = 86.78^***^, *df* (1, 385)				
*Model 2: Predicting psychological trauma symptoms (PTS)*	*β*	*SE (HC4)*	*t*	*95% CI*	
				*[LL,*	*UL]*
Psychological inflexibility	.28^***^	.04	5.32	[.13,	.28]
Avoidance coping	.40^***^	.05	7.57	[.30,	.52]
Model statistics	*R*^*2*^ = .33, *Adj R*^*2*^ = .33, *F*(HC4) = 86.41^***^, *df* (2, 384)				
*Model 3: Predicting perceived threat (PT)*	*β*	*SE (HC4)*	*t*	*95% CI*	
				*[LL,*	*UL]*
Psychological inflexibility	.21^***^	.06	4.01	[.13,	.38]
Avoidance coping	.28^***^	.09	5.32	[.30,	.65]
Model statistics	*R*^*2*^ = .18, *Adj R*^*2*^ = .18, *F*(HC4) = 39.18^***^, *df* (2, 384)				
*Model 3: Predicting non-adherence*	*β*	*SE (HC4)*	*t*	*95% CI*	
				*[LL,*	*UL]*
Psychological inflexibility	.11^*^	.05	2.16	[.01,	.20]
Avoidance coping	.17^**^	.07	2.90	[.07,	.35]
Psychological trauma symptoms	.18^***^	.07	3.34	[.09,	.35]
Perceived threat	.22^***^	.04	4.39	[.09,	.23]
Model statistics	*R*^*2*^ = .26, *Adj R*^*2*^ = .25, *F*(HC4) = 32.04^***^, *df* (4, 382)				
*Indirect effects*		β	Boot SE	Boot 95% CI	
				LL	UL
Total indirect effects		.24	.03	[.17,	.31]
PI → AC → non-adherence		.08	.03	[.03,	.14]
PI → PTS → non-adherence		.05	.02	[.02,	.09]
PI → PT → non-adherence		.05	.02	[.02,	.08]
PI → AC → PTS → non-adherence		.03	.01	[.01,	.06]
PI → AC → PT → non-adherence		.03	.01	[.01,	,05]

*Note:* ^***^p < .001, ^**^p < .01; ^*^p < .05; N = 387; *β* = Standardized regression coefficient

Adj R^2^ = Adjusted R^2^ (computed using two online calculators: https://www.danielsoper.com/statcalc/calculator.aspx?id=25 and https://www.analyticscalculators.com/calculator.aspx?id=25)

The mediational analysis examined the chain mediation effects of avoidance coping, psychological trauma symptoms, and perceived threat; as well as the double mediation paths (a) through avoidance coping and psychological trauma symptoms and (b) through avoidance coping and perceived threat. The results reveal that all indirect paths were significant. That is, the indirect effect of psychological inflexibility on non-adherence to the preventive behavioral measures was significant through avoidance coping (*β* = .08, *Boot 95% CI* [.03,.14]), psychological trauma symptoms (*β* = .05, *Boot 95% CI* [.02,.09]), and perception of threat (*β* = .05, *Boot 95% CI* [.02,.08]). These findings support hypotheses 2a, 2b, and 2c. The double mediation paths were also confirmed. Specifically, the indirect effect via avoidance coping and psychological trauma symptoms (*β* = .03, *Boot 95% CI* [.01.06]) and via avoidance coping and perception of threat (*β* = .03, *Boot 95% CI* [.01,.05]). Therefore, hypotheses 3a and 3b are supported.

## Discussion

The study assessed the role of psychological inflexibility, avoidance coping, perceived threat, and psychological trauma symptoms in the uptake or adherence to the preventive behavioral measures among refugees in Uganda during the COVID-19 pandemic. The study reveals significant findings with several implications for theory and practice. Results relating to the covariates show that gender had substantial effects on avoidance coping and psychological trauma symptoms among refugees. Notably, men reported higher usage of avoidance coping and psychological trauma symptoms than women. Previous research on gender differences relating to war revealed that women were more likely to suffer and exhibit trauma symptoms [55,56]. Concerning avoidance coping, previous research has revealed contradictory findings when it comes to gender variations. Whereas some studies show that women tend to use avoidance coping [57], the study by Eschenbeck [58] found that boys tend to use avoidance strategies, more than girls, to cope with stress. In this study, the high levels of avoidance coping and psychological trauma symptoms among men could also be accounted for by a culture that requires men to exhibit greater emotional strength; hence, they are less likely to seek social support regarding their psychological and emotional problems, resulting in poor mental health [59].

Consistent with the hypothesis, psychological inflexibility had significant positive effects on non-adherence, and the hypothesized serial mediation model explained 26% of non-adherence to preventive behavioral measures. Whereas the serial mediation model was significant, psychological inflexibility also had substantial direct effects on non-adherence to the preventive behavioral measures. Beyond situational avoidance and inflexible attention, psychological inflexibility encompasses other core processes, such as disrupted values and inaction [27], which have the potential to directly thwart behavioral change and lead to non-adherence to behavioral control measures. Levin et al. [60] propose that problem behaviors that underlie some psychological disorders tend to develop through avoidant and inflexible adjustment strategies. This proposition is that psychological inflexibility activates avoidance behavior, hindering effective behavior change. In this direction, the results suggest that psychological inflexibility was an essential factor in refugees’ non-adherence to the preventive behavioral measures aimed at slowing the spread of COVID-19 virus.

The results show that psychological inflexibility was positively associated with psychological trauma symptoms. Previous research suggests that psychological inflexibility hinders recovery from psychological trauma, given that inflexibility reduces the likelihood of value-based actions and taking advantage of contingencies available in one’s environment [18,20,32] and rigidity in adjustment [60]. Hence, refugees with high levels of psychological inflexibility and who have experienced potentially traumatic experiences may develop psychological trauma symptoms. The study of Gray et al. [27] revealed that refugees with high levels of psychological flexibility have higher chances of developing traumatic stress symptoms. For the present study, the average stay in Uganda at the time of data collection was 4.61 years, indicating that many refugees had only recently arrived at the beginning of the COVID-19 pandemic. Newly arrived refugees tend to have high levels of trauma [61,62] associated with pre-migration and asylum-seeking processes, as well as the struggle to adjust to the new yet largely poor living conditions th in the refugee settlements in less developed countries. Therefore, the high levels of anxiety and worry over COVID-19 contagion could have exacerbated psychological trauma symptoms [63,64].

Additionally, the results revealed that psychological inflexibility has a positive effect on the perception of threat. It is expected that the avoidance and inflexible attention associated with psychological inflexibility could have inhibited the ability to perceive threats and risks related to the pandemic. However, the disruptive nature of the pandemic, including the rapid spread of the virus and related deaths globally, meant that even the avoidance and maladjustment attributes that characterized psychological inflexibility could not deter individuals from recognizing the threat of COVID-19. Nonetheless, the negative implications of inflexibility for behavioral adjustment can be seen in the positive associations between psychological inflexibility and perceived threat with non-adherence to preventive behavioral measures. These positive correlations suggest that although individuals recognized the risks of contracting COVID-19 and its severe consequences, their perception of these risks did not translate into effective adherence behavior.

Relatedly, regarding the role of avoidance coping, the results showed a positive relationship between avoidance coping and psychological trauma symptoms, perceived threat, and non-adherence to the preventive behavioral measures among refugees. There is an overlap between avoidance coping and PTSD that has been discussed in extant literature [65]. Importantly, avoidance behavior is an indicator and antecedent of psychological trauma [65]. Avoidance coping involves strategies such as denial [49], which may limit the ability to effectively evaluate the threat, thereby limiting the motivation to cope [66]. In addition, strategies such as behavioral disengagement, self-blame, and venting have the potential to exacerbate symptoms of psychological trauma and limit the ability for behavioral adjustment [67].

Results showed that the hypothesized serial and double mediation model was supported. First, all three mediator variables (avoidance coping, psychological trauma symptoms, and perceived) mediated the relationship between psychological inflexibility and non-adherence to preventive behavioral measures. Moreover, the double mediation paths through avoidance coping strategy and psychological trauma symptoms, and through avoidance coping strategy and perception of threat were also significant. These results suggest that perceived threat and psychological trauma played a magnifying role in non-adherence to the preventive behavioral measures. Therefore, high psychological inflexibility had higher chances of not adhering to the preventive measures because the inflexibility predisposed them to maladaptive coping strategies, given that inflexibility hinders flexible coping strategies [60].

The study has some challenges that may limit the generalizability of our findings. Firstly, the restrictions on movement and physical interactions at the time of data collection made it impossible to use probabilistic sampling approaches. Therefore, convenient samples are used. However, this was based on location sampling, which is seen as appropriate for disaster-related research [62]. In this direction, we collected data from only one rural and one urban settlement (out of 13 known refugee settlements in the country). In addition, the selected settlements are occupied mainly by South Sudanese and Somali refugees. Future studies could focus on a broader range of refugee populations. Secondly, prolonged subsequent lockdowns hindered the possibility of conducting follow-up studies. Therefore, the study used cross-sectional data collected using self-report measures. Hence, there is a likelihood of the effect of common method bias [68,69]. The challenge of self-reports can be addressed by using objective tools to measure behavior. For example, Sheynin [19] proposes avoidance tasks that can be used to assess avoidance coping behavior. In the context of refugees with low levels of education, self-reports on quantitative rating scales may not yield very accurate data. Therefore, qualitative studies or triangulation or data collection approaches may be more appropriate to generate more robust and reliable data. An additional challenge associated with cross-sectional data is the lack of temporal precedence, making mediational analysis problematic [70]. Therefore, we cannot conclude with certainty about the role of psychological inflexibility, avoidance coping strategy, perception of threat, and psychological trauma symptoms in lowering refugees’ adherence to preventive behavioral measures.

The study was conducted among refugees in resource-constrained settings. The precisely defined target population constrains the generalization of the findings. However, beyond the precariousness of refugees’ living conditions, these study variables are critical for understanding behavioral responses to crises. Therefore, the findings can help understand how refugees and individuals in constrained settings might respond during adversity. For example, psychological inflexibility and psychological trauma are generally associated with psychological and behavioral adjustment difficulties among refugees [65,71–73]. These have similar effects in populations other than refugees [35,74–76]. Likewise, the perceived threat influenced health behavior and behavioral adjustment during the COVID-19 pandemic [77,78]. Thus, despite the above limitations, the findings can help understand and shape the behavioral responses of refugees and other vulnerable populations in times of crisis and disasters.

In addition to the generalizability of the findings discussed above, our study has implications for practice. Psychological inflexibility is posited in the ACT model as a determinant of several psychological dysfunctions, while the opposite, psychological flexibility, is seen as essential to good mental health and functioning [74,79–81]. Therefore, psychosocial interventions for refugees should incorporate aspects of the ACT model. For example, mindfulness training interventions effectively improve psychological flexibility, mental health, and behavioral adjustment [79,81,82]. Such interventions are not only helpful in dealing with psychological and behavioral challenges related to the pandemic but also the general mental health problems that refugees often experience, such as anxiety, stress, depression, and PTSD [83,84]. Specific actions, such as mindfulness training, can be implemented to boost flexibility and adaptive coping [85–87], thereby improving refugees’ psychological states and fostering behavior change in situations where behavior adjustment is required.

The results have also highlighted the role of perceived threat in adherence to preventive measures. Whereas a decrease in psychological inflexibility and using adaptive coping are essential in enabling accurate assessment of threats posed by stressful situations, extant evidence shows that awareness programs were also critical in the case of COVID-19 [88,89]. However, refugees are often excluded from such programs due to language barriers. Therefore, awareness programs calibrated explicitly to the needs and context of refugees could help foster behavioral change and adherence in similar situations, especially those that require behavioral adjustment.

## Conclusion

The results of the current study have revealed that psychological inflexibility was a strong determinant of refugees’ uptake of preventive behavioral measures devised to control the COVID-19 pandemic. The serial and double mediation analyses revealed that psychological inflexibility was correlated to non-adherence to preventive behavioral measures via each of the mediating variables, including avoidance coping, perception of threat, and psychological trauma symptoms. Avoidance coping tendencies affected the perception of threat and psychological trauma symptoms, resulting in non-adherence. The results suggest that psychological inflexibility impairs coping, consequently heightening psychological trauma symptoms in stressful situations and diminishing the ability to accurately perceive the threats or risks posed by the stressful situation. Therefore, reducing psychological inflexibility (and thereby boosting flexibility) could be essential for coping with and enhancing behavioral adjustment to control pandemics and other related health challenges.
